# Editorial: ICT for Assessment and Rehabilitation in Alzheimer’s Disease and Related Disorders

**DOI:** 10.3389/fnagi.2016.00006

**Published:** 2016-01-20

**Authors:** Philippe Robert, Iracema Leroi, Valeria Manera

**Affiliations:** ^1^CoBTeK Laboratory (Cognition, Behaviour, Technology), University of Nice Sophia Antipolis, Nice, France; ^2^Centre Mémoire de Ressources et de Recherche, CHU de Nice, Nice, France; ^3^Institute of Brain, Behavior and Mental Health, University of Manchester, Manchester, UK

**Keywords:** information and communication technology, aging, Alzheimer’s disease, serious games, virtual reality, assessment, rehabilitation, online education

**The Editorial on the Research Topic**

**ICT for Assessment and Rehabilitation in Alzheimer’s Disease and Related Disorders**

As depicted by the cover image, this special issue unites seemingly disparate items and objects. However, importantly, all these items are related to the theme, “ICT for Assessment and Rehabilitation in Alzheimer’s Disease and Related Disorders.” Under this rubric, our special issue addresses four subtopics: assessment, rehabilitation, virtual reality (VR), and education.

In the last decade, there has been a growing interest in employing information and communication technologies (ICTs) with the aging population, as well as with people living with Alzheimer’s disease (AD) and related disorders. This interest has manifested in several ways. For example, there has been a notable increase in the amount of research funding dedicated to projects related to the theme of ICT and aging at the level of private, national, and international funding agencies. Furthermore, there has been a significant growth in the number of academic journals and publications dedicated to the topic with a resulting explosion in the number and quality of research, policy, and clinically related publications. Finally, there has been a welcome proliferation of national and international conferences/workshops themed on ICT and aging, as well as strong representation in the topics and themes of the most important AD- and dementia-related conferences.

One of the peculiarities, and indeed fascinating aspects, of this domain is that it is intrinsically interdisciplinary and is dependent on the tight collaboration among professionals from diverse backgrounds, including researchers, clinicians, engineers, and business people. Those involved are driven by the thrill of innovation and the potential to apply exciting new technologies to some of the most basic aspects of older people’s daily lives. The ultimate aim of these endeavors is to improve quality of life, enhance independence, and promote healthy living in the older population. Yet this work can be accompanied by complications, snags and hitches, not the least of which is the necessity for all the players to develop a common language. The need to span the boundaries among disciplines has resulted in some unique professional pairings, such as is exemplified by the recently launched team of CoBTek, the Cognition, Behavior and Technology Unit of the University of Nice Sophia Antipolis, which has arisen from the collaboration of the Nice Memory Clinic and INRIA. CoBTek is now working with “Innovation Alzheimer” Association in France to host and deliver annual interdisciplinary workshops devoted to developing recommendations for the use of ICT in people with AD and related disorders (Robert et al., 2013; Robert et al.). The inspiration behind the present research topic was one of the successful outputs of the workshops and was intended to bring together international researchers and professionals working in this new field to provide new insights into the current use of ICT in healthy and pathological aging. The aim also included the need to identify challenges and new perspectives in the field, gather recommendations for the application of ICT in AD and related disorders in clinical practice, and showcase cutting edge clinical research.

## Ict for Assessment

As summarized by König et al., ICT can help in the assessment and evaluation of patients’ behavioral and functional impairments, and thus should be more consistently employed in clinical trials. ICT allow the development of non-invasive methods to facilitate an early patient diagnosis and to evaluate more objectively behavioral and functional deficits and their evolution over time. Importantly, this assessment and evaluation can take place in a more realistic, “ecologically”-valid setting, and thus enhance the validity of the findings. Along the same theme, Lyons et al. described an innovative in-home system for continuous evaluation employing pervasive computing technologies (including passive motion sensors, contact sensors, monitoring of computer use, and phone monitors). The system was designed to prospectively and continuously detect and record health-related parameters, such as gait and mobility, sleep and activity patterns, and medication adherence in older people, with the aim of detecting early signs of cognitive and physical decline. Similarly, König et al. presented a study in the context of the FP7 Dem@Care project where they employed video-analysis methods to automatically recognize performance in activities of daily living in elderly people with MCI and early AD. Results confirmed that automated video-analysis can provide objective and quantitative measures of autonomy and functional impairment in ADLs. Other ICT-based methods that can be applied in the assessment of people with AD and related disorders include actigraphy, computerized-testing, automated audio-analysis techniques, and instruments able to quantify objectively specific biological markers, such as eye movements and EEG signals. Crawford et al. offer a good example of how eye tracking can be used to monitor saccadic eye movements of people with AD, throwing light on the importance of monitoring the evolution of participants’ performance over time. Finally, Wen et al. reviewed the coupling and synchronization EEG analysis methods for the evaluation and early diagnosis of MCI, and outlined differences between the two methods, underscoring their advantages and disadvantages.

## ICT for Training and Rehabilitation

Information and communication technology is increasingly taking on the role of training and rehabilitation of physical and cognitive functions in people with dementia, as well as fostering social interactions and emotional well-being. In the present issue, Lancioni et al. demonstrated that a computer-mediated verbal reminiscence program could significantly improve reminiscence in people with moderate AD. In a similar vein, Valentí Soler et al. showed that robot-based therapeutic sessions in nursing homes and daycare centers reduced apathy and irritability in residents with dementia.

Another growing domain is exemplified by serious games (SGs). Manera et al. presented a new SG developed within the FP7 VERVE program, which trains executive functions in people with MCI and AD. The feasibility study showed the overall acceptability of the game among those with MCI and early AD, as well its potential role in training. Despite the increased interest in the field and the promising results, SG must be validated in larger and better controlled clinical trials, as suggested by Muscio et al. Specifically, it would be important to define standard SGs parameters and to combine and harmonize different outcome measures, including recognized and validated biomarkers.

## Virtual Reality

Virtual reality is another emerging field that is successfully seeping into clinical settings. VR has been applied and adapted by researchers and clinicians working with elderly people for assessment as well as training and rehabilitation. The usefulness of VR tasks for the early assessment of cognitive impairment is elegantly illustrated by Tarnanas et al., who employed a VR-based SG targeting spatial navigation and executive functions combined with a dual-task walking measurement. Interestingly, they were able to establish that the combination of motor and cognitive performance parameters was more reliable than cognitive performance alone for the early characterization of amnesic MCI, a state which in some cases may be a prodrome of AD. In a similar vein, Serino et al. used a VR-navigation task requiring the encoding and memorization of spatial representations, and found that participants had a specific deficit in the ability to encode and store allocentric (viewpoint-independent) representation.

Despite these promising examples, more work needs to be done to exploit the full potential of VR. In their mini-review, García-Betances et al. demonstrated that only a handful of studies, thus far, have utilized fully immersive VR systems, which use 3D displays that virtually place the person inside the virtual environment. In contrast, the majority of VR-based studies used non-immersive or semi-immersive VR, with lower levels of immersion and interaction. This is unfortunate, as immersion and interaction are features that may strongly have impact on the quality of the assessment and the effectiveness of training in clinical populations. For instance, Jebara et al. showed that in healthy older adults, the possibility to choose the itinerary to follow in a VR-navigation task improved episodic memory encoding compared to a passive navigation condition.

## ICT for Education, Communication, and Caregiver Support in Dementia

Several clinical trials are starting to investigate the potential of the Internet and online tools to create interactive educational programs to support caregivers of people with dementia. The aim here is to minimize caregiver burden and stress and improving quality of life. Internet-based approaches may also facilitate communication among the different stakeholders involved in a dementia care network. For example, Span et al. tested an interactive web tool developed to enhance shared decision-making among people with dementia, caregivers, and health-care professionals. They found that all the stakeholders found the tool useful for decision making and fostering communication.

Finally, another, and seemingly “space-age,” approach to supporting caregivers is outlined in the paper by Pino et al. Here, the advantages of ICT are being exploited in an attempt to reduce ­caregiver burden by using socially assistive robots (SAR). The authors explored the opinions and attitudes toward SAR of healthy elderly persons those with MCI, as well as caregivers of people with dementia. They found that although SAR are perceived as useful solutions, especially by participants experiencing current needs (MCI and caregivers), it is clear that the field has a still a long way to progress.

As editors, we would like to extend our sincere gratitude to all our authors who have contributed to this exciting and ground-breaking special issue of Frontiers.

## Author Contributions

PR, VM, and IL edited the papers and wrote the manuscript.

## Conflict of Interest Statement

The authors declare that the research was conducted in the absence of any commercial or financial relationships that could be construed as a potential conflict of interest.
